# Supplementary material for: Analysis of Genetic Diversity of Fescue Populations from the Highlands of Bolivia Using EST-SSR Markers
Source: Genes (Basel). 2022 Dec 8;13(12):2311. doi: 10.3390/genes13122311 (PMC9777978; doi:10.3390/genes13122311)
Supplement: Supplementary file 1 [file genes-13-02311-s001.zip › Table S1.pdf]

Table S1. Populations of *Festuca* analyzed in the present study and the geographic locations and altitudes of the seed collection sites in the highlands of Bolivia.

Population	Department	Site	Geographic location		Altitude	Biogeographic province
			LAT (S)	LON (W)		
1	Oruro	Caracollo	17 39 20.3S	67 13 36.5W	3771	Xerophytic Puna
2	Oruro	La Joya	17 42 26.2S	67 16 10.4W	3770	Xerophytic Puna
3	Oruro	Condoriquiña	17 31 43.2S	67 15 14.5W	3957	Xerophytic Puna
4	Oruro	Callipampa	18 26 21.0S	66 58 19.5W	3715	Xerophytic Puna
5	Oruro	Sora Sora	18 09 33.1S	66 59 11.2W	3757	Xerophytic Puna
10	Oruro	Chilliwani	17 37 51.6S	67 13 45.6W	3799	Xerophytic Puna
24	Oruro	Illapaya	17 38 55.7S	67 11 55.3W	3777	Xerophytic Puna
25	Oruro	Caracollo	17 36 18.7S	67 15 57.2W	3839	Xerophytic Puna
35	Oruro	Toraca baja	18 09 59.6S	66 59 39.0W	3742	Xerophytic Puna
11	La Paz	Comanche	16 51 35.5S	68 25 46.0W	3970	Mesophytic Puna
14	La Paz	Puerto Perez	16 17 30.5S	68 34 00.6W	3829	Mesophytic Puna
15	La Paz	Igachi	16 17 41.3S	68 33 08.5W	3834	Mesophytic Puna
16	La Paz	Achica arriba	16 46 35.0S	68 10 52.6W	3927	Mesophytic Puna
17	La Paz	Querani	16 13 39.1S	68 30 22.4W	3971	Mesophytic Puna
18	La Paz	Chirioco	16 23 49.3S	68 22 01.2W	3929	Mesophytic Puna
19	La Paz	Orkojipiña	16 31 44.2S	68 20 53.7W	3862	Mesophytic Puna
20	La Paz	El Tholar	16 59 29.4S	68 04 28.4W	3979	Mesophytic Puna
21	La Paz	Sica Sica	17 19 05.1S	67 46 02.7W	3906	Mesophytic Puna
26	La Paz	Comanche	16 57 28.4S	68 25 23.9W	4037	Mesophytic Puna
27	La Paz	Konchamarca	17 22 29.8S	67 27 27.5W	3987	Mesophytic Puna
28	La Paz	Konchamarca	17 22 29.7S	67 27 28.8W	3981	Mesophytic Puna
13	La Paz	Chirapaca	16 17 59.5S	68 30 14.1W	3885	Mesophytic Puna
29	Potosí	Kucho Ingenio	19 52 52.3S	65 40 58.9W	3659	Xerophytic Puna
30	Potosí	Janko Huaje	19 49 40.4S	65 35 57.0W	3918	Xerophytic Puna
31	Potosí	Janko Huaje	19 50 48.2S	65 34 15.3W	3577	Xerophytic Puna
32	Potosí	Cerro rico	19 37 25.2S	65 44 02.2W	4316	Xerophytic Puna
33	Potosí	Totorapampa	19 26 44.5S	65 51 26.9W	3566	Xerophytic Puna
34	Potosí	Tambo Alcalá	19 19 34.6S	66 02 04.1W	4072	Xerophytic Puna
6	Cochabamba	Tiraque	17 27 23.9S	65 43 09.8W	3217	Mesophytic Puna
7	Cochabamba	Sankayani alto	17 25 32.9S	65 37 41.9W	3930	Mesophytic Puna
8	Cochabamba	Laguna alta	17 24 08.4S	65 36 58.2W	4073	Mesophytic Puna
9	Cochabamba	Toralapa	17 28 50.3S	65 37 08.9W	3673	Mesophytic Puna
12	Cochabamba	Cruce Tiraque	17 23 51.9S	65 51 58.6W	3689	Mesophytic Puna
22	Cochabamba	Cumbre	17 41 18.8S	66 47 04.4W	4325	Mesophytic Puna
23	Cochabamba	Melga	17 25 33.2S	65 54 57.4W	3338	Mesophytic Puna
36	Cochabamba	Villa Junin	17 24 31.4S	65 42 45.3W	3406	Mesophytic Puna
37	Cochabamba	Boqueron Khasa	17 29 16.7S	65 36 41.5W	3641	Mesophytic Puna
38	Cochabamba	Vacas	17 31 03.0S	65 36 48.2W	3790	Mesophytic Puna
39	Cochabamba	Normal Vacas	17 31 59.2S	65 36 12.8W	3751	Mesophytic Puna
40	Cochabamba	Salida Vacas	17 33 06.8S	65 36 19.9W	3585	Mesophytic Puna
41	Cochabamba	Sunjani	17 10 12.6S	66 21 08.4W	3974	Mesophytic Puna

42	Cochabamba	Peñas	17 09 20.6S	66 25 57.2W	4193	Mesophytic Puna
43	Cochabamba	Parque Tunari	17 16 15.9S	66 19 49.7W	3809	Mesophytic Puna

LAT (S) = latitude (south of Equator), LON (W) = longitude (west of Meridian).
